# A review of Covid-19 and acute kidney injury: from pathophysiology to clinical results

**DOI:** 10.1590/2175-8239-JBN-2020-0204

**Published:** 2021-05-28

**Authors:** Inah Maria D. Pecly, Rafael B. Azevedo, Elizabeth S. Muxfeldt, Bruna G. Botelho, Gabriela G. Albuquerque, Pedro Henrique P. Diniz, Rodrigo Silva, Cibele I. S. Rodrigues

**Affiliations:** 1 Universidade Estácio de Sá, Curso de Medicina, Rio de Janeiro, RJ, Brasil.; 2 Universidade Federal do Rio de Janeiro, Hospital Universitário Clementino Fraga Filho, Rio de Janeiro, RJ, Brasil.; 3 Pontifícia Universidade Católica de São Paulo, Faculdade de Ciências Médicas e da Saúde, São Paulo, SP, Brasil.

**Keywords:** Coronavirus Infection, Acute Kidney Injury, Cytokines, Inflammation Mediators, Review, Mortality, Morbidity, Infecção por Coronavírus, Lesão Renal Aguda, Citocinas, Mediadores de Inflamação, Revisão, Mortalidade, Morbidade

## Abstract

Acute kidney injury (AKI) in hospitalized patients with COVID-19 is associated with higher mortality and a worse prognosis. Nevertheless, most patients with COVID-19 have mild symptoms, and about 5% can develop more severe symptoms and involve hypovolemia and multiple organ dysfunction syndrome. In a pathophysiological perspective, severe SARS-CoV-2 infection is characterized by numerous dependent pathways triggered by hypercytokinemia, especially IL-6 and TNF-alpha, leading to systemic inflammation, hypercoagulability, and multiple organ dysfunction. Systemic endotheliitis and direct viral tropism to proximal renal tubular cells and podocytes are important pathophysiological mechanisms leading to kidney injury in patients with more critical infection, with a clinical presentation ranging from proteinuria and/or glomerular hematuria to fulminant AKI requiring renal replacement therapies. Glomerulonephritis, rhabdomyolysis, and nephrotoxic drugs are also associated with kidney damage in patients with COVID-19. Thus, AKI and proteinuria are independent risk factors for mortality in patients with SARS-CoV-2 infection. We provide a comprehensive review of the literature emphasizing the impact of acute kidney involvement in the evolutive prognosis and mortality of patients with COVID-19.

## Resumo

A lesão renal aguda (LRA) em pacientes hospitalizados com COVID-19 está associada a maior mortalidade e um pior prognóstico. No entanto, a maioria dos pacientes com COVID-19 tem sintomas leves e cerca de 5% podem desenvolver sintomas mais graves e envolver hipovolemia e síndrome de disfunção de múltiplos órgãos. Em uma perspectiva fisiopatológica, a infecção grave por SARS-CoV-2 é caracterizada por numerosas vias dependentes desencadeadas por hipercitocinemia, especialmente IL-6 e TNF-alfa, levando à inflamação sistêmica, hipercoagulabilidade e disfunção de múltiplos órgãos. A endotelite sistêmica e o tropismo viral direto às células tubulares proximais renais e podócitos são mecanismos fisiopatológicos importantes que levam à lesão renal em pacientes com infecção mais crítica, com uma apresentação clínica que varia de proteinúria e/ou hematúria glomerular a LRA fulminante, exigindo terapias renais substitutivas. Glomerulonefrite, rabdomiólise e drogas nefrotóxicas também estão associadas a danos renais em pacientes com COVID-19. Assim, a LRA e a proteinúria são fatores de risco independentes para mortalidade em pacientes com infecção por SARS-CoV-2. Fornecemos uma revisão abrangente da literatura, enfatizando o impacto do envolvimento renal agudo no prognóstico evolutivo e na mortalidade de pacientes com COVID-19.

## Introduction

The advent of severe acute respiratory syndrome coronavirus 2 (SARS-CoV-2) in Wuhan, China, and the global dissemination of the disease caused by the virus, COVID-19, imposes challenges to health systems around the world. Until mid-November 2020, there were more than 58,900,000 confirmed cases worldwide and almost 1,400,000 people deceased due to the disease^1^. In Brazil, more than 6,000,000 cases and 169,000 deaths were confirmed until November 2020^2^.

The clinical spectrum of the disease ranges from typical and atypical symptoms of upper respiratory tract infection to more severe complications such as pneumonia and acute respiratory distress syndrome (ARDS), which usually requires intensive care. Other complications include heart failure, circulatory shock, and acute kidney injury (AKI)^3^. From a pathophysiological perspective, authors hypothesize that critical COVID-19 is associated with immune dysregulation, cytokine storm, and systemic inflammation. Thus, besides direct viral damage to tissues, organ involvement in COVID-19, such as kidney injury, might be secondary to inflammation, endothelial dysfunction, and hypercoagulability^4^.

Comorbidities such as hypertension, diabetes mellitus, obesity, coronary artery disease, congestive heart failure, arrhythmias, and chronic obstructive pulmonary disease seem to be independent predictors of higher in-hospital mortality in patients with COVID-19^5^^-^ 7. Furthermore, there is growing evidence that patients with chronic kidney disease (CKD) have a higher risk of developing severe forms of COVID-19 and increased mortality, raising concerns for this group of patients^8^^-^ 14. The clinical peculiarities, evolution, and prognosis of patients with CKD and COVID-19 will not be discussed in this article. Nevertheless, the assessment of CKD and other comorbidities as potential risk factors for COVID-19-induced AKI is vital to characterize the clinical profile of patients who develop kidney injury during the course of SARS-CoV-2 infection.

Regarding renal complications in patients with COVID-19, an initial retrospective case study from China reported an AKI incidence of around 13.1%, raising initial concerns regarding kidney involvement associated with COVID-19 infection^15^. Therefore, clarifying the pathophysiological mechanisms of kidney injury by SARS-CoV-2 through kidney biopsies is imperative to more precisely define the spectrum of renal disorders associated with COVID-19^16^^,^ 17.

Based on the growing evidence correlating AKI with SARS-CoV-2 infection and the background previously cited, the authors postulate the following hypotheses: (1) AKI induced by SARS-CoV-2 infection is possibly multifactorial, associated with direct viral aggression to renal parenchyma and hyperinflammation induced by COVID-19, being imperative to elucidate the balance between direct viral cytotoxicity and systemic inflammation. (2) Due to the theorized association with immune hyperactivity and cytokine storm, AKI might be more prevalent in patients with more severe disease and ARDS, correlating with a higher need for intensive care and mechanical ventilation. (3) AKI is possibly associated with higher mortality and a worse prognosis in patients with COVID-19. Furthermore, it is vital to assess the association between the degree of kidney injury, potential recovery of renal function upon resolution of the infection, and development of kidney sequelae post-COVID-19.

**Figure 1:**
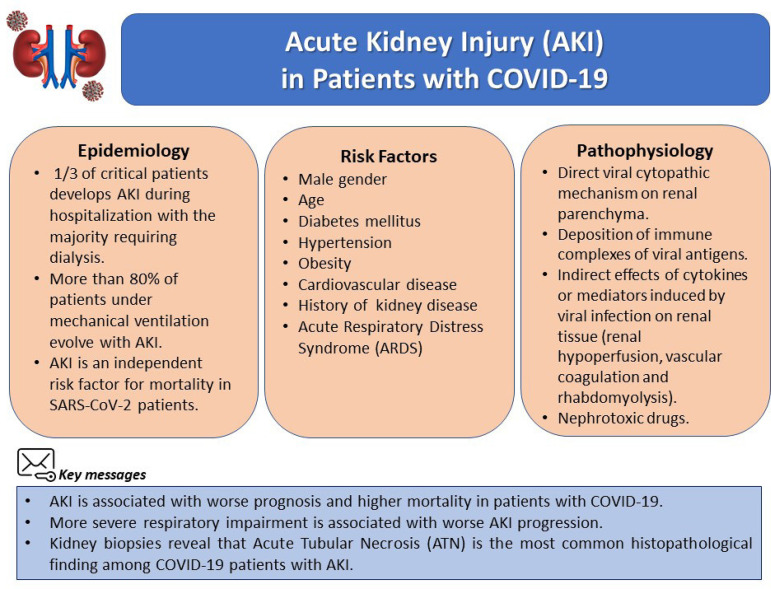
Acute kidney injury in patients with COVID-19. Brief summary of the key points regarding AKI development in COVID-19 patients.

Considering the significant morbimortality presented by the occurrence of COVID-19 evolving with AKI, the present review aims to aggregate the latest evidence regarding AKI in patients with COVID-19 (Figure 1).

## Methodology

A thorough literature scoping review based on the PubMed electronic bibliographic database was performed between April and November 2020, using the following Mesh terms: "Renal", "Kidney", "Acute kidney injury", and "COVID-19", with adoption of PICO strategy and classification of the level of evidence.

The guiding question to construct the review was: what is the latest scientific evidence regarding AKI in critically ill and hospitalized patients with COVID-19. Authors included prospective studies, retrospective studies, meta-analysis, systematic reviews, clinical trials, and clinical guidelines regarding AKI and COVID-19. Articles unrelated to the central theme were excluded from the revision. Articles that were not in English were also excluded. After exclusion, 83 articles were selected for the construction of the present article and were cited directly or via cross-reference in the review hereby exposed.

In the absence of specific anti-SARS-CoV-2 treatments, we will not discuss management with supportive care and the use of renal extracorporeal therapies for critically ill patients with evidence of kidney involvement.

## Integrated Discussion

### COVID-19 AND AKI - PATHOLOGY

#### Pathophysiology (Table 1)

The AKI development due to COVID-19 is presumed to be due to a hyper inflammatory state, triggered by viral infection, possibly associated with viral cytopathic mechanism^18^.

**Table 1:** Summary of the main pathophysiological mechanisms of acute kidney injury in patients with COVID-19

Origin of Kidney Injury	Pathophysiological Mechanism
Viral Cytopathic Effect	The strong association between SARS-CoV-2 and RAAS suggests a direct viral aggression towards the kidney parenchyma, culminating in kidney injury. SARS-CoV-2 through Spike (S) surface protein could bind with ACE-2, expressed in kidney tissue, facilitating viral entrance and direct injury to the kidney parenchyma. ACE-2 is mainly expressed in the apical brush border of proximal tubular cells and podocytes. Early studies from autopsies of post-mortem kidney tissues revealed through electronic microscopy the presence of possible spherical viral particles in tubular epithelium and podocytes, suggesting that SARS-CoV-2 viral tropism might directly affect the kidney. Nonetheless, more robust data from recent autopsy studies were not able to detect viral particles in immunohistochemistry and in situ hybridization, causing the hypothesis of viral replication in renal parenchyma controversial.
ARDS	Studies emphasize a pathological axis between AKI and ARDS. Despite unclear pathophysiology, authors hypothesize mechanical ventilation, hypoxemia, and systemic inflammation as major mechanisms. High pressure ventilation may not only cause lung injury but also systemic inflammation and organ dysfunction due to cytokine release syndrome. Moreover, higher PEEP values is associated with hypercytokinemia and AKI. Positive pressure ventilation can decrease cardiac preload and induce kidney hypoperfusion. In the context of ARDS, hypoxia and hypercapnia is also associated with inflammation leading to AKI, which can enhance inflammation leading to alveolar cells apoptosis and increased vascular permeability, diminishing pulmonary function, and characterizing an ARDS-AKI pathological axis.
Cytokine Storm/ Inflammation	SARS-CoV-2 infection, mainly in severe forms, is associated with immune hyperactivity, hypercytokinemia, and systemic inflammation. Systemic inflammation is associated with multi-organ endotheliitis. Therefore, endotheliitis leading to endothelial dysfunction and complement activation causes hypercoagulability, microangiopathy, altered renal blood flow, hypoperfusion, ischemia, and kidney injury. Therefore, hypercytokinemia associated with COVID-19 might lead to severe impairment of renal microcirculation. Moreover, authors also postulate that kidney injury induced by SARS-CoV-2 can also be indirectly associated with immune mechanisms triggered by viral renal cellular damage, as inflammatory cytokines originated from macrophages and complement-mediated mechanisms from viral cytopathic kidney cell injury can aggravate tubular and interstitial injury. Cytokine storm and immune dysregulation prompts immune-mediated kidney injury. APOL1 gene expression may possibly play an important role in the pathogenesis of AKI due to its important association with inflammation and viral infection.
Myocardium/ Acute Heart Failure/ Hemodynamic Status	Studies are also describing viral tropism of SARS-CoV-2 to ACE-2 receptors present in the myocardium, causing major left ventricular systolic function depression and consequent hemodynamic impairment which can lead to kidney hypoperfusion and AKI. Additionally, right ventricular dysfunction secondary to PTE or pulmonary hypertension associated with hypoxia and/or hypercapnia can also cause hemodynamic instability in patients with COVID-19. Volume depletion and inappropriate volume resuscitation associated with kidney hypoperfusion can aggravate kidney injury.
Rhabdomyolysis	Histopathological studies have demonstrated rhabdomyolysis with histologic evidence in patients with COVID-19. Rhabdomyolysis is associated with massive release of myoglobin into systemic circulation, with myoglobinuria, cast formation, and iron deposition in proximal tubular cells causing intratubular obstruction. Direct toxicity on kidney tubular cells causes acute tubular necrosis.

Abbreviations: RAAS, renin-angiotensin aldosterone system; AKI, acute kidney injury; ARDS, acute respiratory distress syndrome; PEEP, positive end-expiratory pressure.

SARS-CoV-2, especially when causing severe disease, induces systemic inflammation, hypercytokinemia, and multiple organ dysfunction syndrome^19^^,^ 20. Immune dysregulation and the consequent inflammatory hyperactivity promote a cytokine storm, predominantly by IL-6, IL-2, and TNF-alpha, resulting in systemic endothelial dysfunction and a state of hypercoagulability^20^^,^ 21.

Therefore, in patients with systemic inflammation and hypercytokinemia, these pathophysiological mechanisms might result in AKI primarily due to intravascular volume depletion, hypotension, and consequential renal hypoperfusion, resulting in pre-renal AKI, or even acute tubular necrosis (ATN)^22^. Cytokine storm may be intimately related to alveolar and tubular damage in patients with ARDS, characterizing a lung-kidney crosstalk^22^.

In a cohort of 41 hospitalized patients, Huang et al. (2020)^23^ found a high prevalence of ARDS (27%) and AKI (7%), reporting that critical patients hospitalized in intensive care units (ICU) presented higher concentrations of IL-10, but similar concentrations of IL-6 when compared to patients without intensive care. Authors claim that kidney tubular epithelium damage generates a higher expression of IL-6, emphasizing that increased serum levels of this inflammatory biomarker correlates with increased alveolar permeability and inflammation. Also, ARDS may result in kidney medulla hypoxia, enhancing tubular injury^23^^,^ 24.

Studies emphasize a strong relationship between SARS-CoV-2 and renin-angiotensin-aldosterone system (RAAS)^25^, establishing that SARS-CoV-2, through spike (S) surface protein, has the ability to bind with angiotensin-converting enzyme 2 (ACE-2), facilitating viral entrance and replication in several tissues, such as the kidney parenchyma. Viral attachment to host cellular receptors enables activation and cleavage of S protein through proteases codified by the transmembrane protease serine 2 gene (TMPRSS2), allowing the fusion between viral and host membranes. Thus, expression of ACE-2 and TMPRSS2 is crucial for the viral invasion of the host cell^26^^-^ 28.

Kissling et al., in a study of sequential analysis of renal cells mRNA, identified a higher co-expression of ACE-2 and TMPRSS in proximal kidney tubular cells and podocytes^16^. Viral podocytopathy and its effects in proximal kidney tubules may possibly result in intrinsic AKI in patients with COVID-19^29^. Despite current reports indicate kidney tubular injury as the most prevalent form of kidney involvement in COVID-19, some studies highlight collapsing glomerulopathy (CG) as another possible kidney manifestation. Authors suggest that a direct toxic viral effect on podocytes and/or virus-induced cytokine injury to podocytes are key pathophysiological mechanisms for CG development in COVID-19. Moreover, genetic susceptibility, particularly the presence of high risk APOL1 genotypes, might play an important role in CG pathogenesis in patients with SARS-CoV-2 infection, especially among individuals of African-descent. APOL 1 gene expression can be upregulated by viral infection and inflammatory diseases, which activates Toll-like receptor-3 and triggers cell injury pathways, enhancing kidney damage^16^^,^ 30.

Su and collaborators reported histopathological and ultrastructural renal findings from autopsies of 26 patients with COVID-19 who died of respiratory failure. Through electronic microscopy, it was possible to observe spherical viral particles, characteristic of SARS-CoV-2, in proximal tubular epithelium and podocytes, associated with podocyte displacement of the glomerular basement membrane. These findings substantiate the pathophysiological hypothesis that SARS-CoV-2 may directly damage tubular epithelial cells and podocytes, determining viral cytopathic effect, resulting in AKI and proteinuria^17^. .Therefore, proteinuria in patients with COVID-19 may be associated with this direct viral cytopathic mechanism resulting in tubular injury and proximal protein reabsorption deficit, or even derivative from a glomerular origin, in patients who develop acute glomerulopathies, such as CG^16^^,^ 17 ^,^ 30.

Viral tropism of SARS-CoV-2 to ACE-2 receptors may also be associated with acute myocardial injury, causing major depression of left ventricular systolic function and consequent hemodynamic impairment. Hence, heart-kidney axis imbalance, particularly in critically ill patients, can promote acute inotropic deficit, causing diminished cardiac output, arterial underfilling, hemodynamic instability, and kidney hypoperfusion, resulting in low glomerular filtration rate and consequent pre-renal AKI, characterizing the cardiorenal syndrome^22^^,^ 31.

Another possible mechanism of AKI is rhabdomyolysis which can occur as an initial presentation of COVID-19 or during any phase of the viral infection. Its occurrence is due to the nephrotoxic effect of massive release of myoglobin into circulation, with myoglobinuria, cast formation, and accumulation of iron in proximal tubular cells, causing intratubular obstruction and ATN^32^^-^ 35.

Authors are hypothesizing that AKI in SARS-Cov-2 infection is more associated with systemic inflammation, endothelial dysfunction, and complement activation rather than direct viral cytopathic effect through ACE-2 tropism. Systemic inflammation is associated with multi-organ endotheliitis, which can lead to hypercoagulability, microangiopathy, renal hypoperfusion, and ischemia^36^^,^ 37. Nevertheless, the role of immune dysfunction and inflammation in the pathogenesis of COVID-19-induced AKI is not completely understood.

Indirect kidney injury by immune-mediated mechanisms associated with viral cytotoxicity is also another hypothesis. Interestingly, Diao et al. (2020)^38^ evidenced through histopathological examination an important presence of SARS-CoV-2 antigens in the cytoplasm of tubular cells, concomitant substantial accumulation of CD68^(+)^ macrophages in the tubule-interstitium region, and C5b-9 deposition on the apical brush border of tubular epithelial cells^38^. Authors accentuate that inflammatory cytokines originated from macrophages, and complement-mediated mechanisms caused by viral cytopathic kidney cell damage are directly involved in the pathogenesis of kidney tubular and interstitial damage in patients with COVID-19^38^^,^ 39. Nonetheless, it is not clear if kidney injury in COVID-19 is caused by direct viral damage and intracellular replication or indirectly by immune and inflammatory mechanisms mediated by cytokine release syndrome and viral cytotoxicity, or even a combination of both. Thus, histopathology evaluation of kidney biopsies of patients with COVID-19 and established AKI is vital for a more precise definition of the major pathophysiological mechanisms involved.

#### Histopathology (Table 2)

Current limited histopathological data from kidney biopsies from COVID-19 patients with renal impairment demonstrates that the most predominant form of kidney damage is ATN^40^. Nonetheless, Kudose et al. (2020)^41^ in an analysis of 14 native kidney biopsy samples from patients with COVID-19 described collapsing glomerulopathy (35%) and acute tubular injury (28%) as the most prevalent forms of kidney injury. Moreover, isolated cases of membranous glomerulopathy, minimal change disease, anti-GBM nephritis, and crescentic transformation of lupus nephritis were also described^41^.

**Table 2:** Summary of the major kidney histopathological findings in patients with COVID-19

Author	N	Major Histopathology Findings
Kudose et al. 2020	17	• 15 patients (88%) developed AKI. • Collapsing glomerulopathy: 5 patients; Minimal change disease: 1 patient; Membranous glomerulopathy: 2 patients; Crescentic transformation of lupus nephritis: 1 patient; anti-GBM nephritis: 1 patient; isolated ATN: 4 patients. • Electron microscopy: Absence of definitive virions within renal cells. Immunohistochemical stains for spike and nucleocapsid proteins for SARS-CoV-2 RNA showed no definitive staining. • Authors highlight that the lack of definitive viral particles observed in kidney parenchyma argues against direct viral tropism as the major pathophysiological mechanism for AKI.
Sharma et al. 2020	10	• Severe AKI requiring RRT (80%), proteinuria (100%). • Thrombotic microangiopathy: 2 patients; Pauci-immune crescentic GN: 1 patient; Global and segmental glomerulosclerosis: 1 patient; Widespread myoglobin casts: 1 patient. All patients presented varying degrees of ATN. • Electron microscopy: Ultrastructural examination showed no evidence of viral particles and immunohistochemical staining of kidney biopsy for SARS-CoV-2 were negative • Authors conclude that ATN was the most common histopathological finding in the kidney biopsies of patients with COVID-19 studied. Similar to findings from Kudose et al., as there was no evidence of SARS-CoV-2 viral particles.
Golmai et al. 2020	12	• All patients had a pathologic diagnosis of acute tubular injury with focal ATN (varied from mild to diffuse). No histopathological evidence of GN, vasculitis, or thrombotic microangiopathy. • Electron microscopy: None of the cases demonstrated viral particles similar to coronavirus morphology. Immunohistochemical assays for SARS-CoV-2 nucleocapsid protein were negative in all cases. Additionally, in situ hybridization performed in four cases were negative. • None of the kidney biopsies evidenced viral cytopathic effects or necrotizing inflammatory changes. • As EM did not detect viral particles, authors hypothesize that COVID-19 induced AKI is more predominantly associated with ischemic ATN, resulting from systemic inflammation and hypoperfusion, resembling septic AKI, rather than viral cytopathic effect.
Santoriello et al. 2020	42	• Predominant histopathological finding: Acute tubular injury. • Glomerular fibrin thrombi: 6 patients (focal). • Electron microscopy: SARS-CoV-2 viral particles were not identified at ultrastructural level. ISH for SARS-CoV-2 RNA was not able to identify definite positivity for viral particles. • Authors observed a mild degree of ATI even in the setting of severe AKI. • Authors conclude that COVID-19 induced AKI is associated with a complex etiology involving ischemia, hypoxia, sepsis-associated factors, and nephrotoxicity.
Xia et al. 2020	81	• 41 (50.6%) patients developed AKI. • Predominant histopathological finding: Acute tubular injury. • All autopsied patients evidenced different degrees of tubular injury and cytoplasmic vacuolization in tubular epithelial cells. • Crystallizations were observed in proximal tubular cells and casts, suggesting drug-induced AKI, and glomerular lesions were not remarkable. • Electron microscopy: Few particles enclosed in vesicles were observed in the cytoplasm of renal proximal tubular epithelial cells. • SARS-CoV-2 viral particles were not detected by immunohistochemistry. • Inflammation-mediated vascular endothelial injury and immune hyperactivity might play a major role in kidney injury and critical COVID-19 disease.

Abbreviations: AKI, acute kidney injury; ATN; acute tubular necrosis; ATI, acute tubular injury; EM; electron microscopy; GN; glomerulonephritis.

Additionally, Sharma et al.(2020)^42^ evaluated kidney biopsy samples from 10 patients with confirmed COVID-19 and AKI, where variable degrees of ATN were present in all biopsy samples. Besides tubular injury, thrombotic microangiopathy, widespread myoglobin casts, pauci-immune crescentic glomerulonephritis (GN), and segmental glomerulosclerosis with characteristics of healed collapsing glomerulopathy were also observed. Ultrastructural examination by electron microscopy revealed no evidence of SARS-CoV-2 particles in the kidney biopsy samples^42^. Thus, these studies reveal that patients with COVID-19 develop a wide spectrum of glomerular and tubular diseases, suggesting that the major mechanism for COVID-19-related kidney injury is an exacerbated adaptive immune response concomitant with cytokine mediated kidney damage^41^^,^ 42.

As already mentioned, current studies postulate that renal involvement in SARS-CoV-2 infection is mostly associated with a complex association of inflammation, ischemia, hypoxia, and sepsis-associated factors rather than a direct viral cytopathic pathogenic mechanism^36^^-^ 42. Golmai et al. in a study assessing postmortem biopsies of 12 patients with COVID-19 and stage 2 or 3 AKI revealed considerable similarities between AKI induced by COVID-19 infection and sepsis-associated AKI. All patients had acute tubular injury with focal ATN varying from mild (involvement of isolated tubules) to diffuse (50% or more of epithelial necrosis). Curiously, there was no evidence of GN, vasculitis, or thrombotic microangiopathy. Electron microscopy examination found no significant glomerular abnormalities, and immunohistochemical assays for SARS-CoV-2 nucleocapsid protein were negative in all 12 patients. Kidney biopsies indicated no evidence of vascular microthrombi or direct viral infection. The authors postulate that COVID-19-associated AKI is probably related with inflammatory dysregulation and hypercytokinemia, resulting in ischemic acute tubular necrosis from systemic infection and kidney hypoperfusion^43^.

Likewise, Santoriello et al. (2020)^44^ evaluating kidney histopathology of 42 patients who died of COVID-19 also demonstrated that the most significant finding was mild acute tubular injury with the absence of diffuse thrombotic microangiopathy or acute GN^44^. Moreover, positivity for SARS-CoV-2 was absent during in situ hybridization, corroborating with the results presented by Golmai et al (2020)^43^. Thus, kidney damage associated with COVID-19 infection possibly results from complex mechanisms triggered in a direct or indirect manner by SARS-CoV-2, with further studies required to elucidate the equilibrium between inflammation and direct viral cytopathic effect^41^^-^ 44.

**Figure 2:**
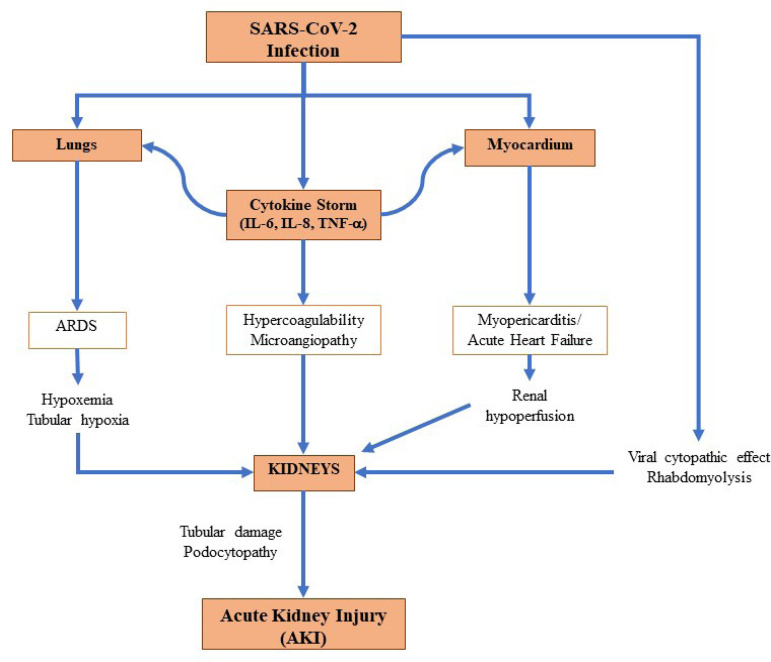
Acute kidney injury pathophysiology in SARS-CoV-2 infection. Brief schematization and summary of the key aspects regarding the pathophysiology of AKI in patients with COVID-19.

Summarizing, AKI in patients with COVID-19 has a multifactorial origin, as previously detailed: (a) direct cytopathic action of the virus on kidney tissue through the angiotensin-converting enzyme 2 (ACE-2) receptor to invade host cells; (b) deposition of immune complexes of viral antigens or specific immunological effector mechanisms induced by viruses; (c) indirect effects of cytokines or mediators induced by the virus on kidney tissue; (d) kidney hypoperfusion, vascular coagulation, hypoxia, shock, and rhabdomyolysis; and (e) direct viral aggression and injury of kidney tubules (Figure 2). ATN is the most prevalent form of kidney injury evidenced in histopathology studies^43^^-^ 47.

#### COVID-19 and acute kidney injury - clinical features and outcomes

AKI emerges as a significant factor associated with worse prognosis in patients infected by SARS-CoV-2. Severe respiratory impairment is associated with a worse progression of kidney injury and, consequently, a poorer clinical outcome^8^^,^ 48. In a preliminary meta-analysis that included 9 studies, the incidence of AKI in hospitalized patients with COVID-19 was 3%. However, in critical patients who needed care in ICU, the incidence level remarkably raised to 19%^49^.

Early Chinese reports evaluating the clinical characteristics and outcomes of patients with COVID-19 alluded AKI as an important adverse outcome but with diverging results. Wang et al. in a case series study describing the clinical characteristics of 138 hospitalized patients with COVID-19 in Wuhan found an AKI incidence of 3.6% and 8.3% in ICU patients^50^. Moreover, Guan et al. described in 1,099 COVID-19 patients a total incidence of 0.5 and 2.9% in patients with severe disease^51^. On the other hand, a retrospective cohort study assessing the clinical characteristics of 113 deceased patients with COVID-19 demonstrated an AKI rate of 25%^52^. The retrospective analysis of 85 patients with laboratory-confirmed COVID-19 in Wuhan from Diao et al. (2020)^38^ and colleagues revealed an AKI incidence of 27%, being more predominant in patients with advanced age (≥ 60 years) (65.22 vs. 24.19%). In the United States, a preliminary case series study describing the clinical characteristics and outcomes of 21 critically ill patients with COVID-19 in the Washington State region revealed an AKI incidence of 19.1%, upholding the data from initial Chinese retrospective studies^53^. Hence, the initial data regarding kidney involvement in COVID-19 described in early case series and retrospective cohort studies raised concerns in nephrologists and clinicians.

Furthermore, Richardson et al. (2020)^54^, in a case series study evaluating the presenting characteristics, comorbidities, and outcomes of 5,700 patients hospitalized with COVID-19 in New York, reported an AKI incidence of 22.2% being more predominant in patients with more advanced age (>65 years) compared to younger individuals (35.5% vs. 25.5%). Hirsch (2020)^(55 )^and collaborators, in another study carried out in New York, raised additional concerns in intensivists and nephrologists regarding kidney involvement in COVID-19. From 5,449 hospitalized patients in 13 hospitals of Northwell Health New York between March and April 2020, 1,993 patients (36.6%) developed AKI and 31.0% were classified with stage 3 AKI. Furthermore, AKI was significantly related to respiratory failure, as 89.7% of patients in mechanical ventilation developed AKI with an in-hospital mortality rate of 35.0%. Amongst patients that did not require ventilatory support, there was a significantly lower incidence of AKI (21.7%)^55^. Interestingly, similar findings were published in a prospective cohort study by Cummings et al. (2020)^56^ where approximately one third (31.0%) of critically ill patients developed severe AKI and required dialysis, exposing, therefore, higher incidence percentages than those previously described in medical literature. Thus, due to the important association between AKI and critical COVID-19, it is imperative to identify and characterize the risk factors for AKI development, clinical manifestations and evolution, prognosis, mortality rates, and risk factors for in-hospital death amongst patients with COVID-19 and AKI.

#### Risk factors for AKI in COVID-19 (Table 3)

Understanding the risk for developing AKI, its clinical association with ARDS, and severe AKI requiring renal replacement therapies (RRT) in SARS-CoV-2 infected patients is vital for patient risk stratification, prognosis, and implementation of preventive and kidney protective measures. A retrospective case series study including 370 hospitalized patients with COVID-19 assessing the incidence and risk factors for AKI development in such patients revealed that hypertension, diabetes, hyperlipidemia, and CKD were correlated with higher odds ratios of AKI development during hospitalization after univariate analysis. Furthermore, mortality was significantly higher amongst patients with AKI compared to patients without AKI (58.1 vs. 19.6%)^57^. Another retrospective cohort analysis including 116 hospitalized patients demonstrated that increased inflammatory biomarkers, decreased glomerular filtration rate, coagulation disorders, and increased markers of cardiac injury, and stress were significantly associated with a higher risk for AKI after univariate analysis^58^. Hence, frontline doctors should closely monitor the kidney function of COVID-19 patients with preexisting comorbidities and/or a laboratory profile denoting a more severe disease due to an augmented risk for AKI development^57^^,^ 58.

**Table 3:** Summary of the major studies regarding risk factors for acute kidney injury in patients with COVID-19

Author	N	Design	Age (years)	Comorbidities	Major findings
Nimkar et al. 2020	370	Retrospective	71 (59-82)	HTN (63.9%) DM (42.5%) DLP (34.9%) CVD (29.9%)	1.Odds for AKI in hospitalized patients with COVID-19 (Multivariable analysis): • African-American race (OR 2.01 [95%CI 1.1-3.6], p=0.02); hyperlipidemia (OR 1.8 [1.04-3.01], p=0.03); History of CKD (OR 3.3 [95%CI 1.4-7.9], p=0.08).
Wang et al.2020	116	Retrospective	62 (55-69)	HTN (40.5%) DM (17.2% CAD (10.3%) CD (6.0%	1.Odds for AKI in hospitalized patients with COVID-19 (Multivariable analysis): • Procalcitonina > 0.1ng/mL (OR 4.822 [95%IC 1.095-21.228], p=0.037); TFGe <60mL/min/1.73m2 (OR= 13.451 [95%IC 1.617-111.891], p=0.016
Hirsch et al.	5.499	Cohort	64 (52-75)	HTN (55.7%) DM (33.0%) CAD (11.0%) HF (6.4%)	1. Risk factors associated with the development of AKI (Multivariate analysis): • Age (OR 1.03 [95%CI 1.03-1.04], p<0.001); Black race (OR 1.23 [95%CI 1.01-1.50], p=0.04); Diabetes (OR 1.76 [95%CI 1.49-2.07], p<0.001); CVD (OR 1.48 [95%CI 1.22-1.80], p<0.001); Mechanical ventilation (OR 10.7 [95%CI 6.81-16.70], p<0.001); Vasoactive drugs (OR 4.53 [95%CI 2.88-7.13], p<0.001).
Cheng et al. 2020	1,392	Retrospective	63 (50-71)	HTN (36.0%) DM (17.0%) CKD (2.0%)	1.Risk factors associated with the development of AKI (Multivariate analysis): • Severe disease (OR 2.25 [95%CI 1.37-3.67]); Higher baseline SCr (OR 2.19 [95%CI 1.17-4.11]); Lymphopenia (OR 1.99 [95%CI 1.12-3.53]); Elevated D-dimer level (OR 2.68 [95%CI 1.07-6.70]).
Fisher et al. 2020	3,345	Retrospective	64.4 (SD 16.4)	DM (27.1%) CKD (12.2%)	1. Risk factors associated with the development of AKI (Adjusted model): • Male (OR 1.6 [95%CI 1.4-1.8]; Non-Hispanic Black ethnicity (OR 1.7 [95%CI 1.3-2.3]). 2. Predictive Model of AKI Stage 2 or 3: • Black race (OR 1.8 [95%CI 1.2-2.8]); Male sex (OR 1.4 [95%CI 1.1-1.7]); DM (OR 1.3 [95%CI 1.0-1.7]); Nursing home resident (OR 1.6 [95%CI 1.2-2.1]); Neutrophil/lymphocyte ratio (OR 1.5 (95%CI 1.2-1.9]); LDH levels (OR 2.1 [95%CI 1.8-2.5]).
Xia et al.2020	81	Retrospective	66.6 (SD 11.4)	HTN (53.1% DM (23.6%) CAD (21.0%)	1. Risk factors associated with the development of AKI: • Age (per 10 years): (HR 1.8 [95%CI 1.2-2.7], p=0.002); Serum IL-6 (HR 1.8 [95%CI 1.2-2.7], p=0.003).

Abbreviations: DM, diabetes mellitus; HTN, hypertension; CVD, cardiovascular disease; CAD, coronary artery disease; CKD, chronic kidney disease; CD,

Previous history of CKD seems to be an important risk factor for severe COVID-19 infection and acute kidney impairment. An observational study in a tertiary care hospital in Milan, Italy evaluating the risk factors of AKI and RRT among 99 invasively ventilated COVID-19 patients evidenced that a significant proportion of patients requiring RRT had preexisting moderate/severe CKD (26.7 vs. 2.9%)^59^. Zhou et al. in a multi-center cohort study including 366 patients with confirmed COVID-19 infection developed a nomogram for predicting the risk of severe COVID-19, where previous history of CKD was an important predictor of disease severity^60^. Therefore, previous history of CKD must be screened during patient triage in patients with confirmed or suspected COVID-19 due to a higher risk of AKI development^59^^,^ 60.

**Figure 3:**
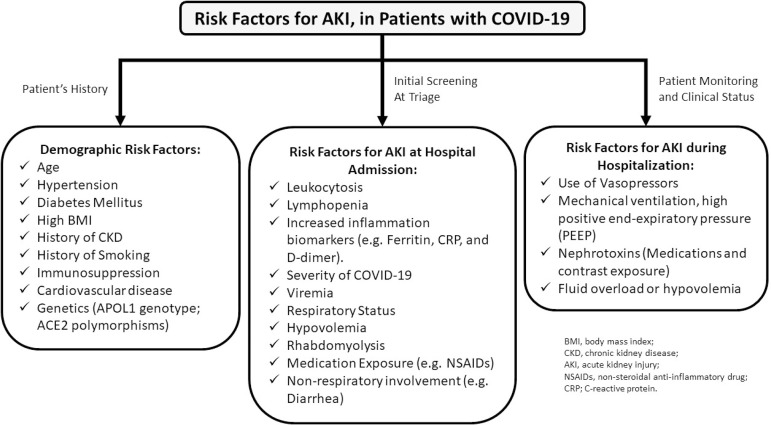
Risk factors for acute kidney injury in patients with COVID-19. Summary of the risk factors for acute kidney injury in COVID-19 according to the consensus report of the 25th Acute Disease Quality Initiative (ADQI).

A consensus report from the 25^(th)^ Acute Disease Quality Initiative (ADQI) Workgroup published in October 2020 suggested that patients must be stratified for risk of AKI based on previous comorbidities and demographics data, where the rationale must be separated in three distinct groups: demographic risk factors, risk factors for AKI at admission, and risk factors for AKI during hospitalization (Figure 3). Therefore, precise risk stratification should guide frontline doctors to monitor and implement preventive and/or precocious therapeutic strategies to benefit high risk patients^61^^,^ 62.

#### Clinical manifestations and laboratory alterations in patients with AKI and COVID-19 (Table 4)

Fisher et al. (2020)^63^, in a retrospective observational study, evaluated AKI incidence, risk factors, and clinical outcomes for 3,345 patients with COVID-19 and 1,265 without COVID-19 hospitalized in New York City compared with a cohort of 9,859 individuals hospitalized a year earlier in the same health system. The primary and secondary outcomes of the study were incident AKI and RRT or mortality, respectively. The incidence of AKI was higher in patients with confirmed COVID-19 compared with patients negative for COVID-19, who were hospitalized during the pandemic, and the historical cohort control (56.0 vs. 37.2 vs. 25.1%). Considering the 1,903 patients with COVID-19 and AKI, 942 (49.5%) were classified as stage 1 AKI, 387 (20.3%) stage 2 AKI, 574 (30.2%) stage 3 AKI, and 28.5% of patients in stage 3 AKI required RRT^63^.

**Table 4:** Summary of the major studies regarding clinical manifestations and laboratory alteration in patients with acute kidney and COVID-19

Author	N	Design	Age (years)	Comorbidities	Major finding
Thakkar et al. 2020	300	Retrospective	AKI: 60 (26-97) Non-AKI: 52 (30-89)	AKI: HTN (69%) DM (448%) Non-AKI: HTN (54%) DM (30%)	**1.Admission laboratory data (Survivors x non-survivors):** -Creatinine (1.1 vs. 1.3, p=0.041); Hemoglobin (13.0 vs. 13.6, p=0.025).. **2. Peak laboratory values:** -Creatinine (3.8 vs. 6.2, p=0.007); Phosphate (7.1 vs. 8.3, p=0.050); Lactate (2.6 vs. 3.4, p<0.01); Procalcitonin (2.6 vs. 6.0, p=0.013); CRP (23.4 vs. 33.8, p=0.001).
Fisher et al. 2020	3.345	Retrospective	64.4 (SD 16.4)	DM (27.1%) CKD (12.2%)	**1.Clinical manifestations:** **(AKI vs. non-AKI)** -Respiratory rate, breaths/min (22.3 vs. 20.6, p<0.001); pulse oximetry (91.7 vs. 94.3%, p<0.001). **2. Initial laboratory data:** -WBC (9.3 vs. 7.8, p<0.001); Neutrophil (7.3 vs. 5.8, p<0.001); neutrophil-lymphocyte ratio (6.6 vs. 4.7, p<0.001); procalcitonin (0.4 vs. 0.2, p<0.001); fibrinogen (659.1 vs. 631.8, p=0.002); CRP (13.2 vs. 7.3, p<0.001); D-dimer (2.2 vs. 1.1, p<0.001); LDH (458 vs. 350, p<0.001); Ferritin (911 vs. 610, p<0.001).
Chan et al. 2020	3.993	Retrospective	64.0 (56.0-78.0)	HTN (38%) DM (26%) CKD (11%) CHF (10%)	**1. Clinical manifestations:** **(AKI vs. non-AKI)** -Temperature (36.9 vs. 37.0); Diastolic BP (125 vs. 124); Heart rate (87 vs. 87); Respiratory rate (20 vs. 18); Oxygen saturation (96 vs. 96). **2. Initial laboratory data:** -WBC (8.7 vs. 6.9); Lymphocyte % (10.6 vs. 15.5); Hemoglobin (12.3 vs. 12.8); Platelets (207 vs. 221); Creatinine (1.42 vs. 0.8); BUN (31 vs. 13).
Xia et al. 2020	3.345	Retrospective	66,6 (±11.4)	HTN (53.1%) DM (23.5%) CAD (21.0%) CD (13.6%)	**1. Clinical manifestations:** **(AKI vs. non-AKI)** -Dyspnea (70.7% vs. 65.0%); Cough (80.5% vs. 75.0%); Fever (82.9% vs. 95.0%); **2. Initial laboratory data:** -Platelets (145.0 vs. 176.5, p=0.03); Lymphocytes (0.50 vs. 0.65, p=0.02); SCr (104.0 vs. 65.5, p<0.001); Elevated SCr (51.2 % vs. 7.5%, p<0.001); Cystatin C (1.74 vs. 1.06, p<0.001); BUN (12.5 vs. 7.1, p<0.001); Serum uric acid (289.5 vs. 164.0, p<0.001); IL-6 (100.4 vs. 36.8, p=0.01); PT (17.2 vs. 16.0, p=0.04); INR (1.40 vs. 1.26, p=0.02); NT-proBNP (1902.0 vs. 843.0, p=0.03).

Abbreviations: DM, diabetes mellitus; HTN, hypertension; CVD, cardiovascular disease; CAD, coronary artery disease; CKD, chronic kidney disease; CD, cerebrovascular disease; CHF, congestive heart failure; CRP, c-reactive protein; SCr, serum creatinine; AKI, acute kidney injury.

From a clinical viewpoint, on hospital admission, patients with COVID-19 and AKI had higher reparatory rates, higher pulse rates, and lower pulse oximetry compared with patients without AKI. The study also demonstrated that patients with COVID-19 and AKI presented increased inflammatory and thrombotic biomarkers when compared with patients without AKI during laboratory evaluation^63^. Data from Pelayo et al. (2020)^64^ upholds these results, as patients with AKI had higher D-dimer levels (5,468 vs. 2,248 ng/mL), revealing an association between kidney injury, systemic inflammation, immune hyperactivity, and a prothrombotic state, observed in more severe SARS-CoV-2 infections evolving with cytokine storm^63^^,^ 64.

Moreover, urinalysis parameters were significantly associated with a higher mortality risk in patients with COVID-19 and AKI. In a prospective cohort study that included 701 hospitalized patients infected with COVID-19, the prevalence of proteinuria and hematuria on hospital admission was 44 and 27%, respectively. Additionally, increased baseline serum creatinine (SCr) and blood urea nitrogen (BUN) was observed in 14.4 and 13.1%, respectively, while 13.0% presented an estimated glomerular filtration rate (eGFR) under 60 mL/min/1.73m^2^. Baseline SCr and BUN, proteinuria, and hematuria were independent risk factors for in-hospital mortality after adjustment for age, gender, severity of disease, comorbidity, and leukocytosis confirmed by Cox analysis model^8^.

A similar pattern was observed in data from Li et al. (2020)^65^ in a multi-centered, retrospective, observational study, including 193 adult patients with laboratory-confirmed COVID-19 in 2 hospitals from Wuhan. Proteinuria was found in 88 patients (60%) and hematuria was identified in 71 patients (48%). Proteinuria was found through semi quantitative analysis as (±) in 31 patients (21%), (+) in 39 patients (27%), (++) in 15 patients (10%), and (+++) in 3 patients (2%), with no significant difference regarding severity and mortality. On the other hand, hematuria was found as (±) in 21 patients (1%), (+) in 21 patients (14%), (++) in 16 patients (11%), and (+++) in 13 patients (9%) with direct correlation with more severe COVID-19. In addition, patients with severe COVID-19 infection presented higher levels of BUN and SCr compared to non-severe patients. After survival analysis with univariate Cox regression model, higher mortality was significantly associated with elevated levels of proteinuria, hematuria, BUN, SCr, and D-dimer. The retrospective analysis also demonstrated that the estimated risk of death in COVID-19 patients with AKI was approximately 5.3 times higher than in patients without AKI^65^.

Moreover, data from Li and colleagues also demonstrated that inflammation and edema of the kidney parenchyma may occur in COVID-19 patients. In 110 COVID-19 patients from Wuhan Tongji hospital with CT scan of the kidney parenchyma, the mean CT value of COVID-19 patients was in the range of 17.0-36.0 HU, with a median of 27.3 HU, being considerably inferior to the controls with no kidney disease (33.2 HU) and patients with pneumonia from a distinct etiology (32.8 HU) admitted to the hospital, corroborating, therefore, the pathophysiological rationale that kidney injury in COVID-19 is associated with an exacerbated inflammatory and immune-mediated reaction^65^.

Studies highlight electrolyte imbalance and proximal tubular abnormalities as important kidney complications associated with COVID-19, with specific laboratory parameters predictors of AKI and disease severity. A retrospective cohort study including 42 hospitalized COVID-19 patients without history of kidney disease identified proximal tubule abnormalities in patients with SARS-CoV-2 infection. Patients were screened for proximal tubulopathy characterized as Fanconi syndrome^66^. The main tubular disorders observed were proteinuria (88%), kidney phosphate leak defined by kidney phosphate threshold/glomerular filtration rate (TmPi/GFR) <0.77 (55%), hyperuricosuria (43%), and normoglycemic glycosuria (43%). Interestingly, patients requiring intensive care presented more frequent tubular abnormalities and were more likely to develop severe Fanconi syndrome. The incidence of AKI was 50% and the tubulopathy proceeded severe AKI stages 2 and 3 in 88% of the patients. The authors hypothesize that acute proximal tubule injury is a predictor of AKI among patients with COVID-19 being also a potential prognostic marker for disease severity, as ICU patients were prone to a more severe proximal tubulopathy^66^.

Werion et al. (2020)^67^, in an analysis of a cohort with 49 patients requiring hospitalization in Belgium, described proximal tubular dysfunction in patients with COVID-19 due to the presence of low molecular weight proteinuria (70-80%), neutral aminoaciduria (46%), and defective handling of uric acid (46%) or phosphate (19%) in these patients, being independent of pre-existing comorbidities, glomerular proteinuria, nephrotoxic medications, or viral load. Data from the Belgium cohort analysis also demonstrated that hypouricemia with inappropriate uricosuria was independently associated with increased disease severity and a remarkable six-fold increase in the risk for ARDS requiring mechanical ventilation^67^.

A meta-analysis and systematic review including 22 observational cohort studies and 17,391 patients assessing kidney complications in hospitalized patients with COVID-19 reported a high incidence of electrolyte disorder. Amongst hospitalized patients, hyperkalemia (12.5%) was the most frequent kidney complication followed by AKI (11.0%), need for RRT (6.8%), and acidosis (5.0%). A single study also reported alkalosis (6.9%) as a significant complication^61^. Thus, in hospitalized patients with risk factors for AKI or established acute kidney impairment, electrolyte imbalance and tubular abnormalities monitoring is crucial^66^^-^ 68.

#### Aki as a risk factor for severe COVID-19 and higher mortality (Table 5)

In addition to worse clinical characteristics and a more adverse laboratory profile, the retrospective analysis from Fisher et al. demonstrated that AKI is also associated with higher ICU admission (20.1 vs. 3.9%) and increased need for mechanical ventilation (29.9 vs. 3.8%) in patients positive for SARS-CoV-2 infection^63^. Additionally, AKI was also associated with higher risk of in-hospital death compared with patients without AKI (33.7 vs. 9.3%), and the prognosis after the establishment of stage 3 AKI was worse in patients with COVID-19. Patients with AKI and COVID-19 presented an increased risk of in-hospital mortality in comparison to negative COVID-19 patients with stage 3 AKI and the historical cohort (52.1 vs. 16.6 vs. 16.6%)^63^. Moreover, observational data from Fominskiy et al. revealed that besides longer duration of mechanical ventilation (15.1 vs. 12.6 days), patients with AKI had a more prolonged time for ICU discharge (16.0 vs. 13.5 days), and increased hospital length of stay (27.5 vs. 25.0 days) in comparison to patients without AKI^59^.

**Table 5:** Summary of the major studies regarding severe COVID-19 and acute kidney injury

Author	N	Design	Age (years)	Comorbidities	Major findings
Thakkar et al. 2020	300	Retrospective	AKI: 60.1 (26.0-97.0) Non-AKI: 52.1 (30.0-89.0)	AKI: HTN (68.75%) DM (44.77%) Non-AKI: HTN (53.94%) DM (30.26%)	**1. Mechanical Ventilation:** -AKI stage: Stage 1 (96%); Stage 2 (91%); Stage 3 (99%). **2.ARDS:** -Severe ARDS: 60% -Severe ARDS and AKI: Stage 1 (21%); Stage 2 (13%); Stage 3 (66%). **3.RRT** -Incidence: 50.8%.
Fisher et al. 2020	3.345	Retrospective	64.4 (SD 16.4)	DM (27.1%) CKD (12.2%)	**1. AKI Incidence** **(COVID-19 positive, negative, and controls)** AKI Stages: Stage 1 (49.5%), Stage 2 (20.3%), Stage 3 (30.2%) -RRT: (4.9% vs. 1.6% vs. 0.9%) -28.5% COVID-19 positive patients with stage 3 AKI required RRT. **2. ICU Admission and Mechanical Ventilation:** 2.1) ICU admission (AKI vs. non-AKI): (20.1% vs. 3.9%, p<0.001). 2.2) Mechanical Ventilation (AKI vs. non-AKI): (29.9% vs 3.8%, p<0.001).
Chan et al. 2020	3.993	Retrospective	64.0 (56.0-78.0)	HTN (38.0%) DM (26.0%) CKD (11.0%) CHF (10.0%)	**1. Clinical Outcomes (AKI vs. non-AKI);** -ICU admission (41% vs. 11%); Mechanical ventilation (44% vs. 6%); Vasopressor use (43% vs. 10%). **2.Predictors Severe AKI:** -Male sex (OR: 1.46 [95%CI 1.2-1.8]); Admission potassium (OR: 1.7 [95%CI 1.6-2.0]); CKD ( OR: 2.8 [95%CI 2.1-3.7]).
Xia et al. 2020	1.752	Retrospective	66.6 (±11.4)	HTN (53.1%) DM (23.5%) CAD (21.0%) CD (13.6%)	**1.Clinical Characteristics ICU (AKI vs. non-AKI):** - APACHE II score (16 vs. 14, p=0.02); SOFA score 7 vs. 6, p=0.03); Coagulopathy (73.2 % vs. 52.5%, p=0.05).
Hirsch et al. 2020	5.499	Cohort	64.0 (52.0-75.0)	HTN (55.7%) DM (33.0%) CAD (11.0%) HF (6.4%)	**1.Mechanical Ventilation** -AKI Stage: Stage 1 (24.2%); Stage 2 (22.0%); Stage 3 (43.5%); RRT (23.2%); AKI any stage (89.7%).

Abbreviations: DM, diabetes mellitus; HTN, hypertension; CAD, coronary artery disease; CKD, chronic kidney disease; CD, cerebrovascular disease; CHF, congestive heart failure; HF, heart failure; ICU, intensive care unit; AKI, acute kidney injury; ARDS, acute respiratory distress syndrome; RRT, renal replacement therapy; SOFA, sequential

Therefore, AKI seems to be associated with a more severe SARS-CoV-2 infection^59^^,^ 63. A meta-analysis and systematic review providing evidence regarding the association between multiorgan dysfunction and COVID-19 demonstrated that AKI was remarkably more prevalent in patients with severe COVID-19 than non-severe disease (OR 10.25 [95%CI 7.60-13.84])^69^. Another meta-analysis with 24 published observational studies including 12,557 patients showed that patients with AKI had a considerably higher severity rate compared with patients without AKI (55.6 vs. 17.7%) and AKI was associated with an outstanding eight-fold increased risk for severe COVID-19^70^. A sub-analysis with a total of 12 studies including 1,968 COVID-19 infected patients, being 551 severe patients and 1,417 non-severe patients, revealed that patients with severe disease had higher levels of Scr. Additionally, another sub-analysis with 7 studies and 1,445 patients correlated severe disease with higher BUN levels. Therefore, AKI is associated with critical illness and increased Scr and BUN levels are potential markers for severe COVID-19^59^^,^ 63 ^,^ 69 ^,^ 70.

Thakkar and colleagues in a retrospective observational study including 300 patients with confirmed SARS-CoV-2 infection requiring intensive care revealed a high incidence of AKI amongst COVID-19 patients in the ICU setting (74.6%). Hospitalized patients who developed AKI were older, with higher body mass index (BMI) values, and with greater prevalence of diabetes mellitus, hypertension, and history of smoking. Concerning kidney outcomes, among 224 patients with AKI, the incidence of AKI stage 1 was 21.8%, while 15.6% evolved with AKI stage 2 and 62.5% stage 3 AKI. Moreover, mechanical ventilation was required in the majority of the patients with AKI, with no significant difference based on stage. From 134 (60%) patients with severe ARDS, 21% had stage 1 AKI, 13% stage 2 AKI, and 66% stage 3 AKI, corroborating the lung-kidney crosstalk pathophysiological hypothesis. The 60-day in-hospital mortality was 66.5% with no difference in mortality based on timing of AKI development or severity. The authors emphasize that the majority of COVID-19 patients with severe AKI requiring intensive care had concomitant moderate to severe ARDS requiring mechanical ventilation^71^.

Hospitalized patients infected with SARS-CoV-2, particularly critical patients, receive a combination of drugs to optimize and stabilize hemodynamic status, prevent or treat opportunistic bacterial and/or fungal infections, and manage thrombotic events with prophylactic or therapeutic anticoagulation therapy, augmenting in a direct or indirect manner, AKI risk. Grein et al. revealed that in a cohort of 61 hospitalized patients with COVID-19 receiving antiviral remdesivir therapy^72^ the drug reduced the median time to recovery (11 vs. 15 days) and mortality (8.0 vs. 11.6%). Nevertheless, patients with severe AKI and end stage kidney disease (ESKD) were excluded from the trial, being important to accentuate that the drug is not recommended in adults with eGFR< 30mL/min/1.73m^2^ due to nephrotoxicity^72^^,^ 73. On the other hand, a preliminary report from the RECOVERY trial demonstrated that in 6,425 hospitalized patients with COVID-19, being 166 (8%) with severe kidney impairment, the use of dexamethasone resulted in a lower 28-day mortality among patients receiving either invasive mechanical ventilation or oxygen alone compared to usual care, being potentially beneficial in critical patients with acute severe kidney impairment and ARDS^74^. Furthermore, antibiotics, which often require adjustments according to kidney function, antifungals, diuretics, among other drugs, could possibly enhance kidney injury in critical patients with COVID-19, emphasizing, in these cases, the necessity of individualized patient approach with a risk-benefit judgement^8^^,^ 48.

As already mentioned, AKI in patients with COVID-19 seems to be also correlated with higher mortality. Xu et al. in a multicenter retrospective study from Wuhan describing the clinical course and predictors of 60-day mortality in 239 critically ill patients with COVID-19 demonstrated a high incidence of AKI, occurring in 119 patients (49.8%). Cox-proportional hazards regression analysis revealed that advanced age (>65 years), thrombocytopenia at ICU admission, ARDS, and AKI independently predicted 60-day mortality in patients with critical COVID-19, thus, kidney involvement might be a common severe complication predictor of greater mortality in the first 60 days of infection in patients with severe COVID-19^75^.

Additionally, Pelayo et al (2020)^64^, in a single-center retrospective study evaluating the clinical characteristics and outcomes of community (CA-AKI) and hospital-acquired AKI (HA-AKI) in patients with COVID-19 in a US inner city hospital system, reported that patients with HA-AKI presented higher rates of in-hospital death (52 vs. 23%, p=0.005) compared with CA-AKI^64^. In a systematic review and meta-analysis including 20 cohort studies and 13,137 hospitalized patients with COVID-19, 77% of patients with AKI had severe infection and was associated with increased odds for mortality (OR 15.27 [95%CI 4.82-48.36]), despite considerable heterogeneity among studies^76^. Furthermore, a cohort of 2,215 adults evaluating the factors associated with death in critically ill patients with COVID-19 in the United States revealed that kidney dysfunction was independently associated with an approximate two-fold increase in the odds ratio for death at ICU admission^77^. Therefore, AKI in COVID-19 seems to be associated with higher mortality risk in hospitalized patients, being also an important risk factor for death in patients requiring intensive care^75^^-^ 77. (Table 6)

**Table 6:** Summary of th e major studies Reding acute kidney injury and mortality in patients wihth COVID-19

Author	N	Design	Age (years)	Comorbidities	Major findings
Thakkar et al. 2020	300	Retrospective	AKI: 60.1 (26.0-97.0) Non-AKI: 52.1 (30.0-89.0)	AKI: HTN (68.75%) DM (44.77%) Non-AKI: HTN (53.94%) DM (30.26%)	**1.Mortality:** -60-day hospital mortality: 66.5% -Mortality Rate RRT: 70.0% **2.Predictors of Risk of In-hospital Mortality:**- -Advancing age, serum potassium levels on admission, and hemoglobin levels on admission.
Fisher et al. 2020	3.345	Retrospective	64.4 (SD 16.4)	DM (27.1%) CKD (12.2%)	**1.Mortality:** -1.1 COVID-19 Positive Cohort -Mortality Rate (AKI vs. non-AKI): (33.7% vs. 9.3%) -1.2 COVID-19 Negative Cohort -Mortality Rate (AKI vs. non-AKI): (13.4% vs. 3.7%) -1.3 Stage 3 AKI and RRT -(COVID-19 Positive vs. Negative) - (52.1% vs. 19.6%; RR 3.8 [CI95% 2.6-3.9]).
Chan et al. 2020	3.993	Retrospective	64.0 (56.0-78.0)	HTN (38.0%) DM (26.0%) CKD (11.0%) CHF (10.0%)	**1.Mortality:** 1.1 Mortality Rates: -In-hospital mortality (AKI vs. non-AKI): (50.0 vs. 8.0, p<0.001). -Mortality Rate AKI (ICU vs. non-ICU): (42.0% vs. 62.0%). -Mortality Rate non-AKI (ICU vs. non-ICU): (7.0% vs. 13.0%). -1.2 Kaplan-Meier Survival analysis (AKI vs. non-AKI): -Survival probability after 30 days: (51.5% vs. 91.8%, p<0.0001). 1.2 Odds for Mortality: -ICU (AKI vs. non-AKI): (OR 11.4 [95%CI 7.2-18]). -All patients (AKI vs. non-AKI): (OR 9.2 [95%CI 7.5-11.3]). -AKI stages (all patients): Stage 1 (OR 4.5 [95%CI 3.5-5.6]); Stage 2 (OR 6.6 [CI95% 4.9-8.9]); Stage 3- no dialysis (OR 20.2 [95%CI 15-27.3]); Stage 3-dialysis (OR 38.7 [95%CI 27.4-54.6]).
Xia et al. 2020	1.752	Retrospective	66,6 (±11,4)	HTN (53.1%) DM (23.5%) CAD (21.0%) CD (13.6%)	**1.Independent Predictors of Mortality:** -IL-6 levels (HR 2.12 [95%CI 1.27-3.53], P=0.004]); Higher levels of D-dimer (HR 1.57 [95%CI 1.13-2.18],p=0.008); SOFA score (HR 1.08 [95%CI 1.01-1.15], p=0.03); Male sex (OR 2.38 [95%CI 1.31-4.33], p=0.004); KDIGO Stage 3 AKI (HR 2.58 [95%CI 1.25-5.31], p=0.010);
Cheng et al. 2020	1.392	Retrospective	63,0 (50,0-71,0)	HTN (36.0%) DM (17.0%) CKD (2.0%)	**1.Mortality:** -1.1 Mortality Rates: -Overall in-hospital mortality: 14% -Overall AKI mortality: 72.0%; Stage 1: 62.0%; Stage 2: 77.0%; Stage 3: 80.0%; 21.0% died 1 day after AKI occurred. -1.2 Odds for Mortality: - AKI KDIGO (OR 5.12 [95%CI 2.70-9.72]).

Abbreviations: DM, diabetes mellitus; HTN, hypertension; CAD, coronary artery disease; CKD, chronic kidney disease; CD, cerebrovascular disease; CHF, congestive heart failure; ICU, intensive care unit; AKI, acute kidney injury; RRT, renal replacement therapy; KDIGO, Kidney Disease Improving Global Outcomes

Thus, these studies alert for the importance of AKI screening in hospitalized patients with COVID-19. Additionally, is imperative to highlight that even in developed countries, such as the United States of America, limited hemodialysis machines and eventual lack of supplies imposes a challenge towards the management of the increased demand of critical patients requiring urgent RRT, engendering a consequential bioethical conflict^55^^,^ 56.

#### Renal replacement therapy and kidney recovery in COVID-19-AKI (Table 7)

Considering the notable incidence and high severity of AKI in hospitalized patients, it is vital to assess RRT need and prognosis, as well as kidney function recovery amongst patients with COVID-19-associated AKI.

**Table 7:** Summary of the major studies regarding renal replacement therapy and renal recovery in patients with AKI and COVID-19

Author	N	Design	Age (years)	Comorbidities	Major findings
Fisher et al.2020	3.345	Retrospective	64.4 (SD 16.4)	DM (27.1%) CKD (12.2%)	**1.RRT (COVID-19 positive vs. negative vs. controls:** -COVID-19 positive vs. negative: (4.9% vs. 1.6%) -COVID-19 positive vs. controls: (4.9% vs. 0.9%) **2.Renal Recovery:** - (42,3% vs. 68,5% vs. 63,9%)
Wilbers et al.2020	37	Retrospective	64 (42-73)	HTN (23.0%) DM (15.0%) CKD (8.0%)	**1.RRT (RRT vs. non-RRT):** -1.1Incidence: 59.0% -1.2 Clinical characteristics: Younger, higher levels of SCr and BUN, Higher incidence of oliguria -1.3 Mortality Rate: (39.0% vs. 44.0%) **2.Renal Recovery:** - -Renal function recovered to KDIGO stage 1 in 64.0% of patients with AKI when discharged from ICU.
Mohamed et al. 2020	575	Observational	AKI: 65 (34-96) Non-AKI 66 (23-97)	AKI: HTN (83.0%) DM (53.0%) Non-AKI HTN (70.0%) DM (47.0%)	**1.RRT and AKI** -1.1 Inflammatory Biomarkers: -AKI vs non-AKI: Ferritin (1016 vs. 680); D-dimer (1.57 vs. 1.13); CRP (163 vs. 93); Procalcitonin (0.37 vs. 0.12); LDH (532 vs. 428). -AKI-RRT: Higher baseline serum ferritin, CRP, procalcitonin, and LDH. Higher D-dimer median peak value (7.8 vs. 3.8, p=0.003). -1.2: Incidence: -Total cohort: 55.0% -ICU: 73.0% -1.3: Risk factors for RRT: -Younger age (61 vs. 68, p=0.0003); Higher BMI (35 vs. 33, p=0.05). -1.4 Mortality: -In-hospital mortality rate: 72.0% -AKI-RRT and MV: 74.0%
Chan et al.2020	3.993	Retrospective	64 (56-78)	HTN (38.0%) DM (26.0%) CKD (11.0%) CHF (10.0%)	**1.RRT:** 1.1 Incidence: -Total: 19.0% -ICU: 32.0% 2.Renal Recovery: -Hospital discharge: 65.0% had recovery of AKI; 35.0% had AKD.
Gupta et al.2020	3.099	Cohort	62(51-71)	HTN (60.3%) DMNID (26.2%) DMID (13.5%) CAD (12.6%) CHF (8.7%) COPD (8.3%)	**1.RRT:** -1.1 Risk Factors AKI-RRT: CKD male sex; non-White race; HTN; DM; BMI; Higher D-dimer; Hypoxemia -1.2 Incidence: -ICU: 20.6% -1.3 Mortality: -Rate: 63.3% -Predictors 28-day mortality AKI-RRT: Older age; severe oliguria. **2.Renal Recovery:** - Hospital discharge (33.9%): 33.8% remained RRT dependent -60 days after ICU admission: 18.1% remained RRT dependent. 1/3 remains RRT dependent on discharge; 1/6 remains 60 days after ICU admission.

Abbreviations: DM, diabetes mellitus; DMNID, diabetes mellitus noninsulin dependent; DMID, diabetes mellitus insulin dependent; HTN, hypertension; CAD, coronary artery disease; CKD, chronic kidney disease; CHF, congestive heart failure; COPD, chronic obstructive pulmonary disease; BMI, body mass index; ICU, intensive care unit; MV, mechanical ventilation; SCr, serum creatinine; BUN, blood urea nitrogen; AKI, acute kidney injury; RRT, renal replacement therapy; KDIGO, Kidney Disease Improving Global Outcomes.

Data from the retrospective analysis from Fisher et al. (2020)^63^ and colleagues also demonstrated that COVID-19 in hospitalized patients was related with a higher need for RRT in contrast to hospitalized patients negative for COVID-19 and the historical control (4.9 vs. 1.6 vs. 0.9%). Nonetheless, patients with COVID-19 and stage 3 AKI requiring RRT remained less RRT-dependent compared with the other two cohorts (5.6 vs. 12.0 vs. 16.4%). However, the authors postulate that the decreased dependency from RRT is directly associated with the high mortality observed in patients with COVID-19 and stage 3 AKI.^56^ Furthermore, patients with AKI and COVID-19 presented less kidney recovery compared with hospitalized patients negative for SARS-CoV-2 infection (42.3 vs. 68.5%) and the control cohort (42.3 vs. 63.9%)^63^.

A systematic review and meta-analysis including 24 studies and 4,963 patients evaluating the prevalence and impact of acute kidney impairment on COVID-19 showed that RRT was required in 5.6% of severe patients, 0.1% of non-severe patients, 15.6% of non-survivors, and 0.4% of survivors.^78^ Another meta-analysis and systematic review of the literature including 142 studies and 49,048 hospitalized patients with positive COVID-19 from the United States and Europe revealed a pooled incidence of AKI and RRT of 28.6 and 7.7%, respectively.^79^ Hence, the results of the meta-analyses and the retrospective analysis from Fisher et al. (2020)^63^ suggest that AKI requiring RRT is associated with hospitalized patients particularly with severe COVID-19 infection^63^^,^ 78 ^,^ 79.

Mohamed et al. (2020)^80^ collaborators conducted an observational study including 575 hospitalized patients with laboratory-confirmed COVID-19 in New Orleans, United States. The incidence of AKI was 28%, where patients with kidney injury presented higher BMI values (34 vs. and 31%), greater incidence of comorbidities, and more exacerbated inflammatory and thrombotic biomarkers compared to non-AKI patients. Significant proteinuria and hematuria were observed in 39 and 19% of patients, respectively. Moreover, 65% of the patients with AKI were admitted or transferred to an ICU and mechanical ventilation, and longer ICU stay, use of vasopressors, and shock were more common in patients with AKI. Interestingly, AKI-RRT represented 55% of the total AKI cohort and RRT was required in 73% of the patients with AKI requiring intensive care, due to volume overload or electrolyte disturbances (hyperkalemia or severe metabolic acidosis). Patients with AKI-RRT had greater BMI, younger age, and higher need for mechanical ventilation compared to patients with AKI not requiring RRT. Additionally, AKI-RRT was associated with higher baseline inflammatory biomarkers and increased median peak D-dimer values in comparison to patients not requiring RRT. AKI was associated with a high in-hospital mortality (72%) with the greatest mortality amongst patients with AKI-RRT needing mechanical ventilation (74%). Data from New Orleans demonstrates a possible linear correlation between inflammation, ARDS, and AKI severity in patients with COVID-19^80^.

In another observational retrospective study including 3,993 hospitalized patients with COVID-19 in New York City, AKI occurred in 1,835 patients (46%) and 347 (19%) required RRT. From a total of 976 patients (24%) admitted to intensive care, 76% developed AKI. AKI in hospitalized COVID-19 patients was correlated with higher ICU admissions, mechanical ventilation, administration of vasopressors, and higher in-hospital mortality (45% vs. 7%). Regarding kidney recovery and prognosis, among 832 discharged patients with AKI, 65% had AKI recovery and 35% presented kidney dysfunction during hospital discharge. Hence, these findings showed that approximately 40% of patients did not present kidney recovery^81^. The retrospective data from Thakkar et al. (2020)^71^ collaborators presented similar findings as 50.8% of patients required RRT and only 30.0% of those patients survived and no kidney recovery was reported. Although 31.5% of patients were discharged from RRT therapy and approximately 70.0% of these patients survived, it is not possible to predict their kidney function in the future and they must be followed by nephrologists^71^.

On the other hand, Wilbers et al. (2020)^82^ in a retrospective analysis investigating mortality and kidney recovery of 37 critically ill patients with COVID-19 requiring RRT evidenced that RRT was not associated with a significant increase in mortality when compared to patients with AKI not requiring RRT. A total of 30 patients were admitted to the ICU and 60.0% developed AKI. Mortality was higher in patients with AKI compared to patients without AKI (41 vs. 20%). Moreover, 22 patients (59%) with AKI required RRT, and patients in the RRT group were younger, presented higher creatinine and BUN levels and higher incidence of oliguria compared to the non-RRT group. Comparing mortality rates between patients with AKI-RRT and AKI not requiring RRT, the mortality rate was slightly higher among patients requiring RRT (44 vs. 39%). Interestingly, kidney function recovery to stage 1 AKI was observed in 64% of patients upon discharge from the ICU^82^.

Likewise, findings from the postmortem kidney pathology evaluation by Santoriello et al. revealed a potential kidney function reversibility upon resolution of SARS-CoV-2 infection. Among the cohort of 33 assessed patients who died from COVID-19, 31 patients developed AKI (94%) being 6 patients with stage 1 AKI (18%), 9 patients with stage 2 AKI (27%), and 16 patients with stage 3 AKI (48%). Moreover, 8 patients with stage 3 AKI (24%) required RRT. Despite high mortality observed in patients with SARS-CoV-2 evolving with kidney impairment, histopathological assessment demonstrated that acute tubular injury was less evident in patients with AKI stage 1. Nonetheless, surprisingly, even in patients developing AKI stage 2 or 3, moderate to severe tubular injury was present in only 29% of the kidney autopsies. Moreover, profound elevation in Scr levels in patients with AKI stage 2 or 3 was associated with mild acute tubular injury in the vast majority of the cases suggesting, despite a high mortality, that there is a potential kidney reversibility among survivors which developed COVID-19-associated AKI during hospitalization^44^^,^ 81.

A multicenter cohort study conducted by the STOP-COVID investigators including 3,099 critically ill adults with coronavirus admitted to intensive care units across 67 hospitals in the United States, evaluated risk factors for AKI-RRT and the 28-day mortality amongst these patients. A total of 637 patients (20.6%) developed AKI-RRT within 14 days of ICU admission. Moreover, AKI-RRT was associated with a high mortality as approximately 55% of AKI-RRT patients died within 28 days of ICU admission. Risk factors for AKI-RRT in patients with COVID-19 were history of CKD, non-white race, hypertension, diabetes, higher BMI, greater D-dimer levels, and more severe hypoxemia during ICU admission. At the end of the 17-day follow-up, the mortality rate was 63.3% and only 216 patients (33.9%) were discharged. Among survivors, 33.8% remained RRT-dependent at discharge and 18.1% remained RRT-dependent 60 days after ICU admission. Besides a high mortality rate in patients with COVID-19 and AKI-RRT, the authors highlight an important RRT dependency incidence in these patients, particularly at discharge^83^^.^ Further research is required to better understand the association between AKI severity and long-term kidney recovery, if it in fact occurs, in patients with COVID-19.

## Conclusion

AKI is associated with more adverse clinical outcomes, worse prognosis, and higher mortality in patients with COVID-19. Preexisting comorbidities such as CKD and increased inflammatory and thrombotic biomarkers are important risk factors for AKI development during hospitalization. Precocious detection of kidney function impairment is imperative to optimize the prognosis and clinical outcomes for these patients.
